# Cardiovascular Protective Effects of Salvianic Acid A on *db/db* Mice with Elevated Homocysteine Level

**DOI:** 10.1155/2017/9506925

**Published:** 2017-09-17

**Authors:** Lei Gao, Parco M. Siu, Shun-wan Chan, Christopher W. K. Lai

**Affiliations:** ^1^Department of Health Technology and Informatics, Hong Kong Polytechnic University, Hung Hom, Hong Kong; ^2^School of Public Health, The University of Hong Kong, Pokfulam, Hong Kong; ^3^Faculty of Science and Technology, Technological and Higher Education Institute of Hong Kong, Tsing Yi, Hong Kong

## Abstract

The onsets of left ventricular hypertrophy (LVH) and endothelial dysfunction (ED) in diabetics, especially in those with elevated homocysteine (Hcy), precede the development of cardiovascular (CV) events. Salvianic acid A (SAA) is a renowned Traditional Chinese Medicine (TCM) that has been applied in the treatment of cardiovascular disease for many decades. In this study, we aimed (1) to investigate the CV protective effects of SAA on ameliorating LVH and ED in *db/db* mice with elevated blood Hcy level and (2) to decipher whether the observed CV protective effects of SAA are associated with Hcy metabolism by modulating the methylation potential and redox status in the liver of the *db/db* mice with elevated blood Hcy level. Our results found that the administration of SAA could significantly slow down the build-up of left ventricular mass and ameliorate ED. Immunological assay analysis on the mouse liver tissue also indicated that SAA treatment on *db/db* mice with elevated Hcy was associated with reduced methylation potential but improved redox status. In conclusion, we revealed that SAA has the potential to protect against the hyperglycemia- and hyperhomocysteinemia-induced oxidative stress on diabetic mice via modulation in Hcy metabolism.

## 1. Introduction

In 2013, 382 million people in the world were living with diabetes mellitus (DM), and the figure is expected to rise drastically to about 600 million by 2035 [1]. There is a growing evidence suggesting that DM is coupled with increased oxidative stress and altered redox state and leads to subsequent onsets of cardiomyopathy [2, 3] and endothelial dysfunction (ED) [4, 5]. Remarkably, diabetic patients are exposed to a higher risk to develop hyperhomocysteinemia (HHcy) when compared to nondiabetic subjects, particularly in those taking metformin antidiabetic medication [6, 7]. According to a previous cohort study in 122 type 2 DM patients, the prevalence of HHcy in type 2 diabetes was 31% [8]. Recent evidences have shown that both long-term and short-term metformin treatments could lead to elevation of Hcy in type 2 DM patients due to reduction of serum folate and Vitamin B12 [9–11]. Today, both hyperglycemia and HHcy status have been recognized as strong cardiovascular disease (CVD) risk factors or predictors [12, 13], and HHcy status was strongly associated with the development of diabetes and diabetic CV complications [11, 14, 15].

Diabetic cardiomyopathy is one of the common diabetic heart complications characterized by left ventricular hypertrophy (LVH), fibrotic changes in the myocardium, and thickening of intramural arterioles in the absence of hypertension and atherosclerosis [16]. Patients with diabetes are well known to have increased risk in the development of LVH owing to the clustering of several CV risk factors, including hypertension, hypercholesterolemia, HHcy, and endothelial dysfunction (ED). It has been reported that HHcy triggers oxidative stress in the heart and leads to cardiac hypertrophy and the subsequent cardiac diastolic function disorder via diminished activity of peroxisome proliferator-activated receptor [17, 18]. Apart from the adverse effects on the heart, extensive clinical and review studies have reported that HHcy status could also impair endothelium-dependent vasodilation in diabetics [19–22]. Interestingly, several previous studies have shown that a short-term Hcy-lowering therapy could improve ED in hypertensive patients [23], and a lower methylation potential (S-adenosylmethionine (SAM) to S-adenosylhomocysteine (SAH) ratio) played a significant role in CVD [24]. These findings therefore suggest that Hcy metabolism might be involved and could serve as a potential treatment pathway to reduce CVD burden in diabetes.

Methylation potential (S-adenosylmethionine (SAM) to S-adenosylhomocysteine (SAH) ratio) and redox status (glutathione (GSH) to glutathione disulfide (GSSG) ratio) are closely linked to Hcy metabolism and are responsible for the clearance of elevated Hcy via the remethylation and transsulfuration pathways. Remethylation converts methionine via SAM and SAH into Hcy in a reversible cycle. Meanwhile, Hcy could also irreversibly transform to GSH via the transsulfuration pathway. As an important intracellular antioxidant, GSH plays a significant role in the redox balance and cell apoptosis [25–28]. GSH reduction was reported as a crucial cause of cardiac cell apoptotic death in diabetic rats, and supplement of GSH could prevent the diabetic rats from heart disease effectively by reversing cell apoptosis and mitochondrial oxidative stress [29]. Generally, SAH level is usually high in subjects with elevated Hcy level because the process of remethylation is a reversible process that catalyzed by SAM and is always at equilibrium due to the high activity of SAH [30]. Also, GSSG is the oxidized form of GSH, and the ratio between GSH and GSSG is fundamental for the maintenance of redox status balance [31, 32].

**Figure 1:**
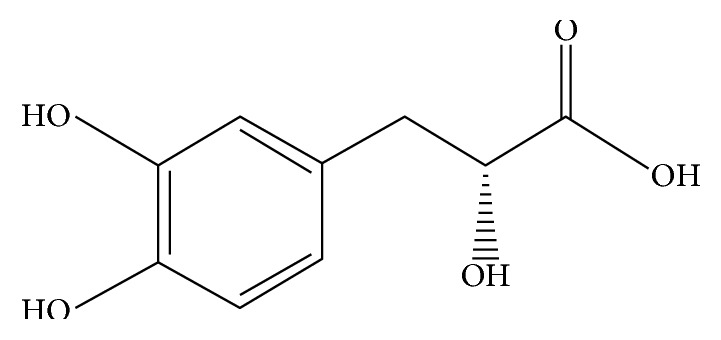
The chemical structure of SAA.

Danshen is a typical Traditional Chinese Medicine (TCM). It is the dried root of *Salvia miltiorrhizae* that has been used in the treatment of CVD for a long period of time [33]. The earliest use of it was first recorded in the books “Shen Nong's Herbal Classic” (First century B. C. - First century A.D. Eastern Han dynasty). According to the TCM theory, Danshen can “remove blood stasis.” Salvianic acid A (SAA), or *Danshensu* with the chemical name of 3-(3,4-dihydroxyphenyl)-2-hydroxypropanoic acid (Figure 1), is one of the major active ingredients in the water-soluble components that extracted from Danshen. It has been proven to possess diverse pharmacological effects such as improving blood circulation, inhibiting platelet adhesion and aggregation, acting against oxidative stress by suppressing the production of reactive oxygen species (ROS), preventing the onset of myocardial ischemia, and ameliorating endothelial cell dysfunction [34–38]. Today, the effects of SAA on ameliorating the progression of LVH and ED are poorly studied, particularly in diabetes with elevated Hcy level. A previous similar study reported that SAA treatment could markedly attenuate HHcy in nondiabetic rats through enhanced production of GSH via activated transsulfuration pathway [39], but whether SAA could exert similar effects on attenuating elevated Hcy level and CVD burdens in a diabetic animal with HHcy is still not clearly understood. Therefore, in the present study, we proposed (1) to investigate the CV protective effects of SAA on ameliorating LVH and ED in *db/db* mice with elevated blood Hcy level and (2) to decipher whether the observed CV protective effects of SAA are associated with the modulation of methylation potential and redox status in the liver of the *db/db* mice with elevated blood Hcy level.

## 2. Materials and Methods

### 2.1. Animals and Intervention

Female diabetic homozygous *db/db* mice and nondiabetic heterozygous *db/m* mice of 10 wks old were housed in the Central Animal Facilities of the Hong Kong Polytechnic University, in a 12 h light/darkness cycle, at a temperature of 20–25°C and humidity of 50–60%. They were allowed regular laboratory chow and tap water ad libitum throughout the experimental period. All the *db/db* mice were then divided into four groups (*n* = 6–9): (G1) DM (control); (G2) DM (SAA); (G3) DM (methionine); and (G4) DM (methionine + SAA). Nondiabetic *db/m* mice (*n* = 10) were grouped as the non-DM group (G5). During the intervention period of 8 wks, all mice were treated according to the following schedule: G1 and G5 received no treatment; G2 received SAA (purity ≥ 98%, Nanjing Zelang Pharmaceutical Technology Co. Ltd., Nanjing, China) at dose of 60 mg·kg^−1^·day^−1^ by gastric intubation; G3 received methionine (purity ≥ 98%, Sigma-Aldrich, St. Louis, Missouri, USA) that was dissolved in tap water as 1% (*w*/*v*), and the water was refreshed every 3 days; and G4 received both SAA (60 mg·kg^−1^·day^−1^ by gastric intubation) and methionine in water (1% (*w*/*v*)). Experimental protocols were performed in accordance with the approved license granted under the Department of Health and approved by the Animal Subjects Ethics Sub-Committee (ASESC) of Hong Kong Polytechnic University.

Fasting blood glucose, echocardiographic, and biochemical parameters were assessed at baseline and at the end of the intervention period. After an overnight fasting for 10 hrs, blood samples were collected from the tail vein of mice at baseline and after intervention for the determination of fasting blood glucose level using a test strip and a glucose meter (Bayer Contour TS, Bayer Inc., Leverkusen, Germany). Then, at the end of an 8 wk intervention, all mice were euthanized. Whole blood was collected by heart puncture and centrifuged at 13,000 rpm in 4°C for 15 min. The serum was then moved to new tubes, snap frozen by liquid nitrogen, and stored under −80°C for later analysis. The liver tissues of the mice were rapidly excised, weighted and washed with phosphate buffered saline, then maintained in centrifuge tubes and snap frozen in liquid nitrogen and stored under −80°C for later analysis. Finally, dissected thoracic aorta tissues were collected carefully and isolated from the surrounding connective tissues and fat and were then cut into aorta rings with a length of around 2 mm for later analysis using myography. This procedure was performed under a dissection microscope with the dissected aorta immersed in Krebs' buffer.

### 2.2. Transthoracic Echocardiography Assessment

**Figure 2:**
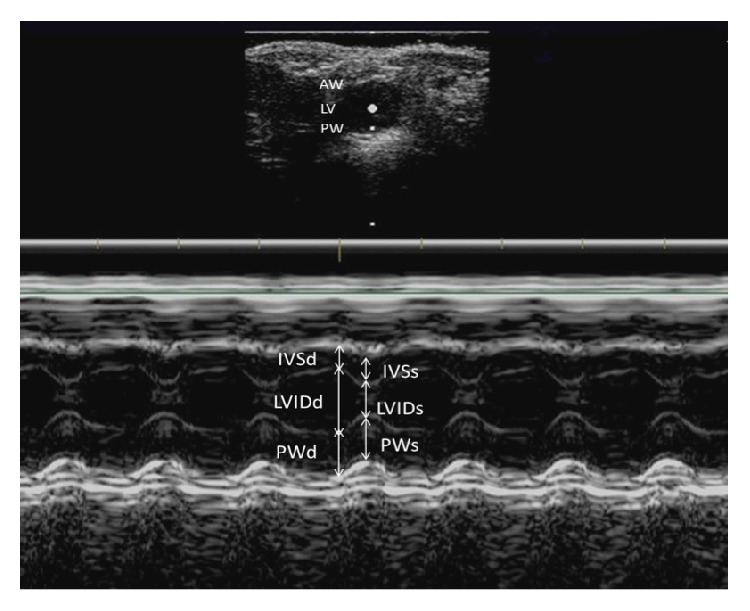
A sample echocardiograph showing the measurement of different parameters of the left ventricle of mice. Upper part of the image showing the cardiac structure of a mouse in parasternal short-axis view at the papillary level in B-mode ultrasound; lower part of the image showing the corresponding left ventricular (LV) and interventricular septal thicknesses (IVS) and LV internal dimensions (LVID) and posterior wall thicknesses (PW) at the diastole and systole of the mice using M-mode ultrasound in a given time period. AW: anterior wall; LV: left ventricular; PW: posterior wall; LVIDd: left ventricular internal dimensions (diastole); LVIDs: left ventricular internal dimensions (systole); PWd: posterior wall thicknesses (diastole); PWs: posterior wall thicknesses (systole); IVSd: interventricular septal thicknesses (diastole); IVSs: interventricular septal thicknesses (systole).

At baseline and the end of intervention, all mice were arranged to undergo transthoracic echocardiographic assessment using an ultrasound system (model: HD11 XE, Philips Medical Systems, Bothell, WA, USA) with a high-resolution broadband compact linear array transducer (model: L15-7io, Philips Medical Systems, Bothell, WA, USA) according to the protocol of our previous work [40]. Mice were anesthetized with an intraperitoneal injection of ketamine (80 mg/kg)/xylazine (10 mg/kg) mixture and rested on a heating pad to maintain their body temperature during the whole scanning procedure. Then, the hair on their ventral thorax was carefully shaved. During echocardiography, the mice were positioned in prone decubitus position, and the cardiac structure in parasternal short-axis view at the papillary level was determined using B-mode. Then, the left ventricular (LV) interventricular septal thicknesses (IVS), LV internal dimensions (LVID), and posterior wall thicknesses (PW) at the diastole and systole were carefully measured using M-mode at the level of the papillary muscles (Figure 2). Finally, LV ejection fraction (EF), LV fractional shortening (FS), and LV mass were calculated using the following [41]:

(1) $$\begin{array}{c} EF\%=100*\left[\frac{{LVIDd}^{3}–{LVIDs}^{3}}{{LVIDd}^{3}}\right],\\ FS\%=100*\left[\frac{LVIDd–LVIDs}{LVIDd}\right],\\ LV\;mass=1.05*\left[{\left(IVSd+LVIDd+PWd\right)}^{3}–{LVIDd}^{3}\right]. \end{array}$$

### 2.3. Endothelial Function Assessment

Immediately after scarification, the mouse aortas were extracted, cut into pieces as aortic rings, and mounted in a Wire Myograph System (610M, Danish Myo Technology, Aarhus, Denmark) using two parallel L-shaped metal prongs that connected with a force transducer. The change in isometric tension of the dissected aortic ring was monitored and recorded by a real-time force acquisition programme, LabChart Pro software (AD Instruments, Sydney, Australia). During the whole procedure, the prepared aortic rings were bathed with 5 mL Krebs' buffer at a temperature of 37°C and with a continuous supply of 95% O_2_ and 5% CO_2_ mixture. After 1 hr of equilibrium, the prepared aortic rings were challenged with 60 mM potassium chloride to confirm its bioactivity. Then, the aorta rings were washed again and challenged by phenylephrine (10^−6^ M) for precontraction until stabilized. Acetylcholine (Ach) at an accumulative concentration from 10^−9^ M to 10^−6^ M was then added to determine endothelium-dependent dilation, and the whole process was then repeated by adding sodium nitroprusside (SNP) at accumulative concentration from 10^−10^ M to 10^−5^ M to determine endothelium-independent dilation. Cumulative concentration-response curves (from 1/10 nM to 100 mM) were constructed, and the vasorelaxation capacity in response to Ach or SNP was expressed as % at different contractions, where 100% relaxation was considered when the active tone had returned to the baseline level. Krebs' buffer, potassium chloride, acetylcholine, phenylephrine, and sodium nitroprusside were all obtained from the Sigma Chemical Company (Poole, Dorset, U.K.).

### 2.4. Biochemical Measurement

Serum Hcy level was determined according to a previous study with minor modification [42]. In brief, 10 *μ*L of serum sample was injected into a high-performance liquid chromatography system which consisted of a Waters 2695 Separation Module (Waters Corporation, Milford, MA, USA), a Waters 474 Detector (Waters Corporation, Milford, MA, USA), and an analytical column (Agilent Zorbax Eclipse XDB-C18, 5 *μ*m, 4.6 × 150 mm, Agilent Technologies, Santa Clara, CA, USA) with a guard column (Waters Symmetry C18, 5 *μ*m, 3.9 × 20 mm, Waters Corporation, Milford, MA, USA). Mobile phase A was set at 50 mM KH_2_PO_4_ (pH = 5.0), and mobile phase B was set at 50 mM KH_2_PO_4_-methanol 1 : 1(*v*/*v*) (pH = 5.0). The flow rate in the column was set at 1.2 mL/min for 10 min. Finally, the concentration of Hcy was determined by measuring the fluorescence signal captured at an excitation wavelength of 385 nm and an emission wavelength of 515 nm.

SAM and SAH levels in liver tissues were determined following the instruction of commercial ELISA kits (item numbers IK00202S and IK00302S, Arthus Biosystems, LLC, Richmond, CA, USA). The SAM/SAH ratio was calculated to denote methylation potential. Glutathione (GSH) and glutathione disulfide (GSSG) levels in the liver tissue were determined using GSH assay kit (item number 703002, Cayman Chemical, Ann Arbor, Michigan, USA). The GSH/GSSG ratio was calculated to denote redox status.

### 2.5. Statistical Analysis

All data were analyzed using IBM SPSS Statistics (version 21.0, IBM SPSS Inc., Chicago, IL, USA) and represented as mean ± SEM. Comparison among the multiple groups was conducted by one-way ANOVA followed by post hoc Bonferroni test, while the mean differences between baseline and post measurements were analyzed by paired *t*-test. A *P* value of less than 0.05 was considered significant. All figures were generated using GraphPad Prism software (version 5.01, GraphPad software Inc., San Diego, CA, USA).

## 3. Results

### 3.1. Fasting Blood Glucose, Body Weight, and Serum Hcy Level

**Figure 3:**
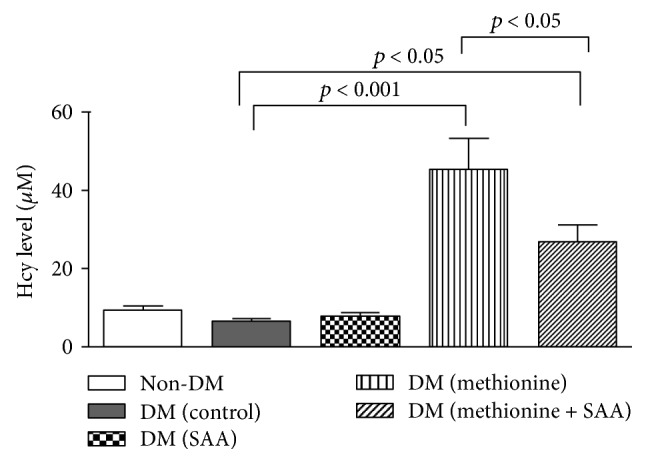
Serum homocysteine (Hcy) level at the end of the experiment. Both DM (methionine) and DM (methionine + SAA) groups exhibit higher Hcy level when compared to the DM (control) group. Besides, the Hcy level in the DM (methionine + SAA) group was significantly reduced to a level lower than that in the DM (methionine) group, probably due to the Hcy-lowering effect induced by the SAA treatment for 8 wks.

All the *db/db* mice exhibited significantly higher fasting blood glucose level and were more obese than the *db/m* mice both at the beginning and at the end of the intervention. However, the fasting blood glucose level in each group was similar before and after 8 wks of intervention period (Table 1). Elevated Hcy levels were successfully induced by using 1% *w*/*v* of methionine in water for 8 wks in the *db/db* mice of the DM (methionine) and DM (methionine + SAA) groups and were evidenced by a 6-fold of increment when compared to the other groups without taking methionine in water (Figure 3). Specifically, consumption of methionine in water significantly elevated the serum Hcy levels in both DM (methionine) group (45.39 ± 7.95 *μ*M) and DM (methionine + SAA) group (26.89 ± 4.17 *μ*M) when compared to the DM (control) group (6.70 ± 0.47 *μ*M) that is taking normal tap water. Besides, the expected elevation of Hcy level after 8 wk of SAA cotreatment in the DM (methionine + SAA) group was significantly reduced when compared to the DM (methionine) group (Figure 3), implicating that DSS has a Hcy-lowering effect on this group. Interestingly, the observed Hcy-lowering effect of SAA was not found in the DM (SAA) group, suggesting that SAA might selectively exert its Hcy-lowering effect on diabetic mice with elevated Hcy level only and have little or no effect on those *db/db* mice with normal Hcy level.

### 3.2. Echocardiographic Assessment

**Figure 4:**
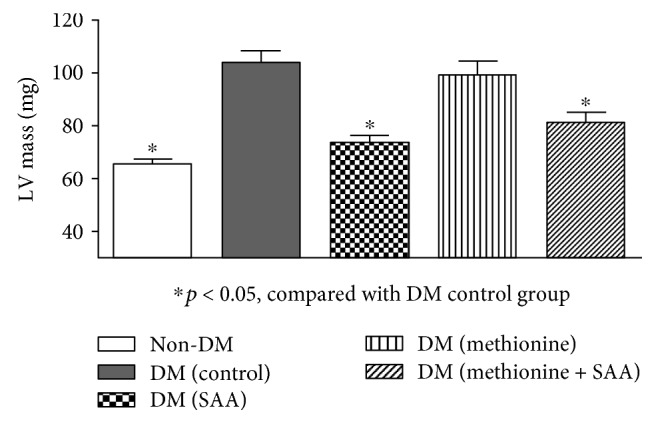
Left ventricular mass (mg) at the end of experiment as estimated by echocardiography. The LV mass in the non-DM, DM (methionine + SAA), and DM (SAA) groups after 8 wks of intervention period was significantly lower when compared to that in the DM (control) group, suggesting that the SAA treatment could delay the progress of the diabetic left ventricular hypertrophy.

We found that the two diabetic groups without taking SAA treatment (DM (control) and DM (methionine)) exhibited increased PWd (58.5%, *P* < 0.01; 58.2%, *P* < 0.05), PWs (31.6%, *P* < 0.05; 32.5%, *P* < 0.05), LVSd (38.1%, *P* < 0.05; 25.4%, *P* < 0.001), and LV mass (49.0%, *P* < 0.001; 31.7%, *P* < 0.01) at the end of intervention period when compared to their baseline values (Table 2). On the contrary, the LV mass in the two SAA-treated diabetic groups (DM (SAA) and DM (methionine + SAA)) was significantly lower than that in the DM (control) group, suggesting a delay in the progression of LVH in the *db/db* mice treated with SAA for 8 wks (Figure 4). Nevertheless, the cardiac function as represented by EF and DS before and after 8 wks was similar in all groups, implicating that SAA might have limited impacts on improving cardiac function, or the intervention period of 8 wks was too short for the potential beneficial effects of SAA on cardiac function to be observed.

### 3.3. GSH, GSSG, SAM, and SAH Levels in Mouse Liver

In the present study, SAM level in the liver was higher in the DM (methionine) group (0.133 *μ*g/g ± 0.021) when compared to the non-DM (0.062 *μ*g/g ± 0.007) and DM (SAA) (0.051 *μ*g/g ± 0.003) groups, while the level of SAH in the liver in the DM (methionine + SAA) group (0.212 *μ*g/g ± 0.024) was found significantly higher when compared to that in the DM (SAA) (0.100 *μ*g/g ± 0.027) and non-DM (0.098 *μ*g/g ± 0.025) groups. Besides, the methylation potential (SAM/SAH ratio) in the DM (methionine + SAA) group (0.46 ± 0.08) was significantly lower when compared to that in the DM (control) group (1.57 ± 0.75). All results were summarized in Table 3.

Meanwhile, GSH level in the livers was lower in the DM (methionine) (3.06 mg/g ± 0.17) and DM (control) (3.04 mg/g ± 0.13) groups when compared to the non-DM group (4.28 mg/g ± 0.18). A higher GSH level was also observed in the DM (methionine + SAA) group (3.77 mg/g ± 0.10) when compared to the DM (methionine) (3.06 mg/g ± 0.17) group. In addition, reduced GSSG level in the liver was observed in both DM (SAA) (0.62 mg/g ± 0.21) and DM (methionine) (0.57 mg/g ± 0.06) groups when compared to the non-DM group (1.14 mg/g ± 0.12). Of note, the redox status as denoted by GSH/GSSG level was significantly improved and elevated in the DM (SAA) group (9.13 ± 2.24) when compared to the DM (control) (4.05 ± 0.47) and non-DM (4.11 ± 0.45) groups. All results were summarized in Table 4.

### 3.4. Endothelial Function Assessment

**Figure 5:**
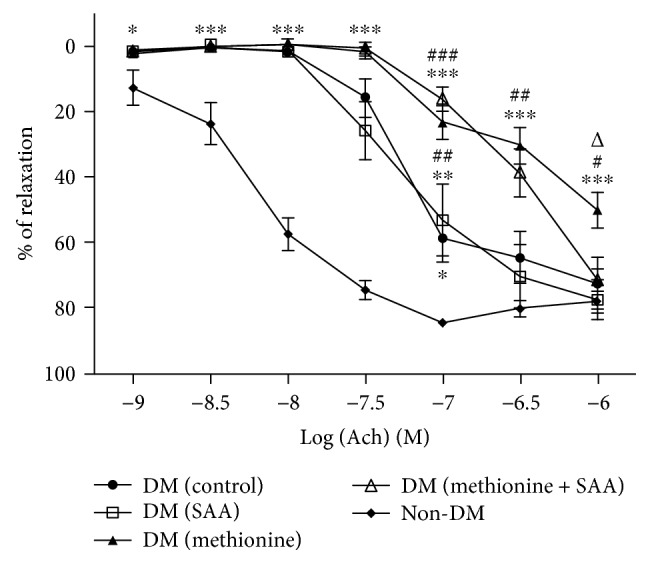
Endothelium-dependent vasorelaxation induced by acetylcholine (Ach). ^∗^*P* < 0.05, ^∗∗^*P* < 0.01, and ^∗∗∗^*P* < 0.001 when compared to the non-DM group; ^#^*P* < 0.05, ^##^*P* < 0.01, and ^###^*P* < 0.001 when compared to the DM (control) group, *^Δ^P* < 0.05 when compared to the DM (SAA) group.

**Figure 6:**
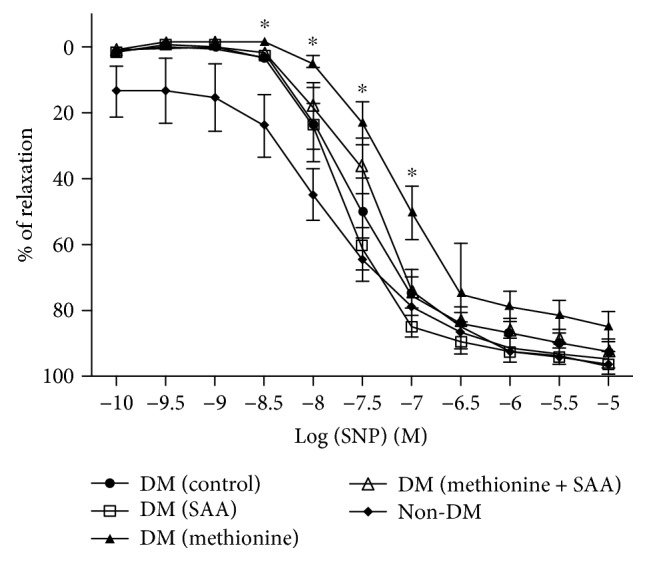
Endothelium-independent vasorelaxation induced by sodium nitroprusside (SNP). ^∗^*P* < 0.05 when compared to the non-DM group.

The results of vascular endothelial function assessments were summarized using concentration-dependent relaxation curves and expressed in Figures 5 and 6. At the concentration of 10^−9^ M–10^−7^ M of Ach, significant reduction in the percentage of Ach-induced relaxation was observed in all the diabetic groups when compared to the non-DM group. From 10^–6.5^M to 10^−6^ M, the reductions in endothelium-dependent relaxations in DM (control) and DM (SAA) were gradually attenuated or improved to an extent similar to the non-DM group. For the DM (SAA + methionine) group, the percentage of relaxation induced by Ach was not improved to the extent similar to the non-DM group until the Ach concentration reached 10^−6^ M. However, the reduction in the percentage of Ach-induced relaxation persists in the DM (methionine) group at all concentrations of Ach. Conversely, the percentage of relaxation induced by SNP in all diabetic groups was similar when compared to that in the non-DM group, except at the concentrations from 10^–8.5^ M to 10^−7^ M where significantly diminished relaxations were observed in the DM (methionine) group when compared to the non-DM group.

## 4. Discussion

Diabetes is a multifactorial disease that leads to various forms of CVD complications. In this study, we collected evidences that SAA, apart from being a potent antioxidant, could possibly serve as a Hcy-lowering drug in diabetes with HHcy and at the same time exert multiple beneficial effects on the cardiovascular system. The present study indicated that oral administration of SAA for 8 wks could delay the progression of LVH and ameliorates ED in *db/db* mice, in particular to those with elevated Hcy level. These observed beneficial cardiovascular effects by SAA in our studied diabetic mice model with elevated Hcy level might be due to an improved redox status by the antioxidant effect of SAA itself and by the increased production of GSH via activated transsulfuration pathway.

Besides, our results indicated that SAA treatment did not significantly affect the serum Hcy level in the diabetic mice with a normal level of Hcy, while clearly lowering down the serum Hcy level in the diabetic mice with elevated Hcy level. This observation might suggest that the beneficial effect of SAA in lowering Hcy level largely depends on the baseline initial value of serum Hcy level of the *db/db* mice. We found that a previous study also exhibited a similar bidirectional Hcy-lowering effect of SAA in the nondiabetic rats [39]. Interestingly, the Hcy level was found lowered in *db/db* mice when compared to *db/m* mice in our study, though it has not reached a significant difference, probably due to (1) a decreased level of methionine in the liver, (2) the sulfur-containing byproduct generated during the methionine metabolism, and (3) a diminished transmethylation activity in the methionine metabolism [43]. Remarkably, as a transgenic diabetic mouse model, *db/db* mice were reported previously as possessing a lower Hcy level when compared to *db/+* mice [43].

## 5. The CV Protective Effects of SAA on Ameliorating LVH and ED in *db/db* Mice with Elevated Blood Hcy Level

As reported in the previous studies, SAA has been indicated to inhibit platelet adhesion and aggregation, act against oxidative stress by suppressing the production of ROS, protect myocardium ischemia, and ameliorate endothelial cells dysfunction via Akt and ERK1/2 phosphorylation [35, 44]. In diabetes, excessive ROS generation could be triggered by hyperglycemia and autoxidation [45]. Elevated ROS was found associated with increased cell death and apoptosis in the heart of the *db/db* mice [46], which led to pathological cardiac remodeling and fibrosis [47] and subsequent abnormal cardiac morphological and functional change [48]. Consistent with a previous study [49], the presence of LVH was found in the DM (control) group, as evidenced by the increased LV mass and wall thickness after 8 wks. With the 8 wks SAA treatment in the DM (SAA) and DM (methionine + SAA) groups, we found the LV mass in these 2 groups was similar to the non-DM group and significantly lower than that in the DM (control) group, suggesting that SAA treatment could prevent or slow down the progression of diabetic LVH (Figure 4).

ED is the initial step in the pathogenesis of atherosclerotic CVD. In the endothelium-dependent vasorelaxation test induced by Ach, the diabetic groups with SAA treatment were found capable to ameliorate the detrimental effect by elevated blood glucose and Hcy in a dose-dependent manner (Figure 5). Besides, the additive detrimental effect of both hyperglycemia and HHcy together with the endothelial function in the DM (methionine) group was evidenced by a relatively lower percentage of Ach-induced vasorelaxation at a concentration of 10^–7.5^ M to 10^−6^ M when compared to the DM (control) group. On the contrary, the endothelium-independent vasorelaxation induced by SNP in all groups were similar at all concentrations of SNP (Figure 6), suggesting that the observed impaired Ach-induced vasorelaxation was due to nitric oxide (NO) bioavailability (i.e., proper function of healthy endothelial cells), not due to NO insensitive (i.e., with adequate NO provided by SNP as NO donor, the vessel could be dilated to the full extent).

## 6. The Methylation Potential and Redox Status in the Liver of the *db/db* Mice with Elevated Blood Hcy Level

As a powerful catechol-O-methyltransferase inhibitor, SAH specifically dominates the methylation of polyphenols, such as SAA [39, 50]. Therefore, a high expression of SAH and low methylation potential would inhibit Hcy methylation and hence limiting the Hcy-lowering capability via a remethylation pathway. In our study, the SAH level in the DM (methionine + SAA) group was significantly higher than that in the DM (SAA) and non-DM groups, while the SAH level in the DM (methionine) group was also higher than that in the non-DM group, though the difference did not reach a significant level. Besides, the SAM level was high in the DM (methionine + SAA) group when compared to the DM (SAA) and non-DM groups. At the same time, the SAM/SAH ratio or methylation potential was also found reduced in the DM (methionine + SAA) group when compared to the DM (control) group. These observations suggested that the methylation capacity in the *db/db* mice with elevated Hcy level was lowered after SAA treatment. Since, we also found that the treatment of SAA could effectively lower down the Hcy level in the DM (methionine + SAA) group by nearly half when compared to the DM (methionine) group (Figure 3). Thus, it is likely that the remethylation process of Hcy has been largely inhibited by the elevated SAH level in the DM (methionine + SAA) group and that the observed Hcy-lowering effect by SAA treatment on the *db/db* mice with elevated Hcy level is most likely through the alternative pathway, that is, the transsulfuration pathway.

Transsulfuration pathway provides an endogenous pathway for the conversion of Hcy into GSH [30, 51]. The significance of the transsulfuration pathway is in the maintenance of the redox homeostasis [52, 53]. In the present study, we found that the GSH level in the DM (SAA + methionine) group was relatively higher when compared to that in the DM (methionine) group after 8 wks of intervention period. This observation further supports our postulation that SAA treatment could normalize Hcy level in the diabetic mice with an elevated Hcy level predominately by activation of transsulfuration pathway and production of more GSH, despite a high expression of SAH and lower methylation potential condition exist after SAA treatment. In addition, the redox imbalance as indicated by reduced GSH/GSSG ratio was significantly elevated in the DM (SAA) group when compared to the DM (control) and non-DM groups, suggesting that SAA is a potent antioxidant. In this study, a higher oxidative stress level was also noted in the *db/db* mice, as indicated by a lower GSH pool size in the DM (control) group, when compared to the non-DM group. In the setting of diabetes, GSH depletion caused by disturbed transsulfuration could lead to the oxidant/antioxidant imbalance on its own, and the impaired transsulfuration could cause the loss of the compensatory GSH synthesis in response to oxidative stress, and lead to further oxidative stress [51, 52].

## 7. Conclusion

From our observations, transsulfuration pathway seems to be activated by SAA treatment and is likely served as a potential target for Hcy-lowering and redox-rebalancing treatments in both hyperglycemic and hyperhomocysteinemic diabetic animals. Although other players in the transsulfuration pathway, such as cystathionine and cysteine levels, were not identified in our experiments, the relatively higher GSH levels in the liver of our diabetic mice with elevated Hcy level after SAA treatment already suggested that SAA could activate the activity of the transsulfuration pathway as a means of lowering Hcy level. We believe that SAA is an important endogenous antioxidant and could work as an exogenous antioxidant against ROS-induced harm in the heart and ameliorate endothelial dysfunction. Besides, the elevated GSH level after SAA treatment provides another explanation for the overall improved antioxidant activity of SAA in protecting against the progression of LVH and ED. Further studies into other key players in the transsulfuration pathway, as well as the underlying mechanism/signaling pathway of CV protective effects by SAA treatment in diabetes with HHcy, are warranted.

## Figures and Tables

**Table 1:** Fasting blood glucose level and body weight at the beginning and end of the experiment.

	DM (control)	DM (SAA)	DM (methionine)	DM (methionine + SAA)	Non-DM
Baseline					
Fasting blood glucose (mmo/L)	27.73 ± 2.08^∗^	22.72 ± 1.50^∗^	27.72 ± 2.14^∗^	23.99 ± 1.71^∗^	5.94 ± 0.50
Body weight (g)	42.95 ± 2.33^∗^	44.63 ± 1.22^∗^	44.76 ± 1.43^∗^	48.42 ± 1.39^∗^	22.14 ± 0.58
After 8 wks					
Fasting blood glucose (mmo/L)	29.18 ± 1.67^∗^	27.08 ± 1.23^∗^	25.77 ± 0.75^∗^	25.64 ± 1.65^∗^	5.43 ± 0.45
Body weight (g)	49.56 ± 4.83^∗^	56.82 ± 2.68^∗^	49.06 ± 3.16^∗^	53.12 ± 2.78^∗^	25.36 ± 0.53

^∗^ *P* < 0.001 when compared to the non-DM group.

**Table 2:** Summary of echocardiography parameters.

Echocardiography parameters	DM (control)		DM (SAA)		DM (methionine)		DM (methionine + SAA)		Non-DM	
	Baseline	Post	Baseline	Post	Baseline	Post	Baseline	Post	Baseline	Post
HR (bpm)	265.78 ± 14.22	249.93 ± 14.23	257.65 ± 12.47	263.32 ± 5.81	263.77 ± 14.00	241.28 ± 7.96	261.90 ± 9.85	238.63 ± 4.75	242.75 ± 6.53	242.26 ± 5.32
*Morphological changes*										
LVIDd (mm)	3.79 ± 0.12	3.54 ± 0.20	3.96 ± 0.12	4.04 ± 0.11	3.80 ± 0.07	3.45 ± 0.22	3.90 ± 0.06	3.75 ± 0.10	3.70 ± 0.07	3.46 ± 0.78^∗^
LVIDs (mm)	2.08 ± 0.05	2.13 ± 0.16	2.34 ± 0.11	2.25 ± 0.11	2.03 ± 0.08	1.97 ± 0.22	2.15 ± 0.09	2.01 ± 0.10	2.16 ± 0.09	2.07 ± 0.09
PWd (mm)	0.53 ± 0.10	0.84 ± 0.09^∗∗^	0.49 ± 0.01	0.53 ± 0.02	0.55 ± 0.04	0.87 ± 0.10^∗^	0.56 ± 0.02	0.67 ± 0.05	0.57 ± 0.03	0.60 ± 0.02
PWs (mm)	0.79 ± 0.04	1.04 ± 0.08^∗^	0.80 ± 0.02	0.87 ± 0.03	0.83 ± 0.04	1.10 ± 0.09^∗^	0.94 ± 0.03	1.03 ± 0.06	0.87 ± 0.04	0.88 ± 0.03
IVSd (mm)	0.63 ± 0.06	0.87 ± 0.07^∗^	0.62 ± 0.02	0.58 ± 0.03	0.67 ± 0.02	0.84 ± 0.04^∗∗∗^	0.65 ± 0.04	0.65 ± 0.03	0.60 ± 0.02	0.65 ± 0.03
IVSs (mm)	1.08 ± 0.05	1.16 ± 0.09	1.06 ± 0.06	1.02 ± 0.05	1.02 ± 0.03	1.16 ± 0.07	1.02 ± 0.04	1.10 ± 0.05	0.96 ± 0.03	1.07 ± 0.04^∗^
LV mass (mg)	69.98 ± 2.54	104.29 ± 4.63^∗∗∗^	71.54 ± 2.27	73.63 ± 2.86	75.66 ± 2.40	99.64 ± 5.30^∗∗^	78.27 ± 2.07	81.65 ± 3.82	67.91 ± 2.07	65.74 ± 1.85
*Functional changes*										
LV EF (%)	83.11 ± 1.29	78.02 ± 2.10	78.77 ± 2.00	82.33 ± 1.42	84.25 ± 1.50	80.68 ± 3.26	83.04 ± 1.35	84.10 ± 1.97	79.72 ± 1.72	78.04 ± 1.89
LV FS (%)	45.11 ± 1.45	40.08 ± 1.86	40.77 ± 2.03	44.30 ± 1.57	46.67 ± 1.71	44.34 ± 3.70	45.08 ± 1.49	46.53 ± 2.08	41.93 ± 1.83	40.23 ± 1.84

HR: heart rate; LVIDd: left ventricular internal dimensions (diastole); LVIDs: left ventricular internal dimensions (systole); PWd: posterior wall thicknesses (diastole); PWs: posterior wall thicknesses (systole); IVSd: interventricular septal thicknesses (diastole); IVSs: interventricular septal thicknesses (systole); EF: left ventricular ejection fraction; FS: left ventricular fractional shortening; LV mass: left ventricular mass. ^∗^*P* ≤ 0.05, ^∗∗^*P* ≤ 0.01, and ^∗∗∗^*P* ≤ 0.001 when compared to its corresponding baseline values.

**Table 3:** The methylation status (SAM/SAH ratio) in the liver of the mice at the end of the experiment.

	DM (control)	DM (SAA)	DM (methionine)	DM (methionine + SAA)	Non-DM
SAM (*μ*g/g)	0.101 ± 0.01	0.051 ± 0.003	0.133 ± 0.021^∗^	0.089 ± 0.011	0.062 ± 0.007
SAH (*μ*g/g)	0.112 ± 0.027	0.100 ± 0.027^∗∗^	0.156 ± 0.010	0.212 ± 0.024	0.098 ± 0.025^∗∗∗^
SAM/SAH ratio	1.57 ± 0.75^∗∗∗^	0.94 ± 0.37	0.86 ± 0.13	0.46 ± 0.08	0.93 ± 0.19

SAM: S-adenosylmethionine; SAH: S-adenosylhomocysteine. All values are expressed as concentration (*μ*g/g) per 1 g of liver tissue. ^∗^*P* = 0.01 versus the non-DM and DM (SAA) groups. ^∗∗^*P* < 0.05 versus the DM (methionine + SAA) group. ^∗∗∗^*P* < 0.01 versus the DM (methionine + SAA) group.

**Table 4:** GSH, GSSG, and oxidative stress (GSH/GSSG ratio) in the liver tissue at the end of the experiment.

	DM (control)	DM (SAA)	DM (methionine)	DM (methionine + SAA)	Non-DM
GSH (mg/g)	3.04 ± 0.13^a,c^	3.53 ± 0.17^b^	3.06 ± 0.17^a,c^	3.77 ± 0.10	4.28 ± 0.18
GSSG (mg/g)	0.85 ± 0.17	0.62 ± 0.21^∗^	0.57 ± 0.06^∗^	0.78 ± 0.07	1.14 ± 0.12
Oxidative stress (GSH/GSSG ratio)	4.05 ± 0.47^∗∗^	9.13 ± 2.24	5.60 ± 0.47	5.20 ± 0.58	4.11 ± 0.45^∗∗^

GSH: glutathione; GSSG: oxidized glutathione. ^a^*P* < 0.001 versus the non-DM group. ^b^*P* < 0.05 versus the non-DM group. ^c^*P* < 0.05 versus the DM (methionine + SAA) group. ^∗^*P* < 0.05 versus the non-DM group. ^∗∗^*P* < 0.05 versus the DM (SAA) group.
